# Noncaloric Sweeteners in Children: A Controversial Theme

**DOI:** 10.1155/2018/4806534

**Published:** 2018-01-08

**Authors:** Samuel Durán Agüero, Lissé Angarita Dávila, Ma. Cristina Escobar Contreras, Diana Rojas Gómez, Jorge de Assis Costa

**Affiliations:** ^1^Escuela de Nutrición y Dietética, Facultad de Ciencias de la Salud, Universidad San Sebastián, Santiago de Chile, Chile; ^2^Carrera de Nutrición y Dietética, Facultad de Medicina, Universidad Andres Bello, Sede Concepción, Talcahuano, Chile; ^3^Escuela de Nutrición y Dietética, Facultad de Medicina, Universidad Andres Bello, Santiago, Chile; ^4^Faculdade de Medicina/FAGOC, Ubá, MG, Brazil; ^5^Universidade Estadual de Minas Gerais (UEMG), Barbacena, MG, Brazil

## Abstract

Noncaloric sweeteners (NCS) are food additives used to provide sweetness without adding calories. Their consumption has become more widespread around the world in all age groups, including children. The aim of this study is to show the state of the art about the intake of noncaloric sweeteners in children, as well as their benefits and consumption risk. Scientific searchers were used (PUBMED, Scopus, and Scielo) to analyze articles that included keywords (noncaloric sweeteners/saccharin/cyclamate/acesulfame potassium/aspartame/sucralose/stevia/children) in English, Spanish, and Portuguese. Authors conclude that it is imperative that health professionals judiciously and individually evaluate the overall benefits and risks of NCS use in consumers before recommending their use. Different subgroups of the population incorporate products containing NCS in their diet with different objectives, which should be considered when recommending a diet plan for the consumer. In childhood, in earlier age groups, this type of additives should be used as a dietary alternative when other forms of prevention in obesity are not sufficient.

## 1. Introduction

Noncaloric sweeteners (NCS) are food additives whose purpose is to provide sweetness without providing calories or glycemic effects; these are used in beverages, foodstuffs, medicines, and even in mouthwashes [1]. The introduction of NCS dates back more than a century [2]. NCS are several magnitudes sweeter than sucrose.

To be able to use NCS they must be approved by international agencies such as Codex Alimentarius (Food Code), FDA, and EFSA [3–5]. Despite these rigorous reviews regarding the safety of NCS, there is still a high level of debate about the use of NCS in food supply, which requires risk assessments to identify potential health problems associated with the consumption of foods containing NCS. A key aspect of the risk assessment of chemical foods is the estimation of dietary exposure. Exposure evaluations require two basic data sources: (1) food consumption and (2) information on the concentration of the chemical product in food. Recent intake assessments have shown that intake of artificial sweeteners usually does not exceed the associated acceptable daily intake (ADI) among many population cohorts [6, 7]. At this time, some studies have presented a probabilistic model to estimate the effect to exposure of food additives, in regard to NCS such as sucralose and acesulfame in children who have taken specialized products in medical nutrition [8].

Saccharin, cyclamate, acesulfame potassium, aspartame, sucralose, and stevia [9] are the sweeteners approved for the consumption. Soft drinks and other sugar-sweetened beverages represent the largest source of added sugars and the discretionary calorie diet for children and adults in the United States and other American countries, considering that 4 of 5 leading drinkers worldwide are found In the region (Chile, Argentina, Mexico, and the United States) [9, 10].

NCS consumption has expanded in South America, as shown by several studies performed in adults [11–13] and children [14–16], especially due to the increasing prevalence of obesity in our region [17].

However, although NCS are safe [18], consumption in children raises controversy both in health professionals and in general public, which has meant that the recommendations for this age group are not similar [19], and in some countries it is even forbidden to recommend its use [20] (references to dietary guidelines or indications of pediatrics in at least 5 American countries). The FDA has set an acceptable daily intake limit (expressed in mg/kg body weight) for each NCS, and this value is usually set at 1/100 of the level with no observed adverse effect (tolerable upper intake levels where there are no observed adverse effects in animal studies) [21]. This opinion provides the use, toxicity, and current guidelines appropriate for the use of NCS in clinical practice.

Most NCS are not metabolized in the body and thus are generally considered safe for consumption. Nevertheless, concern about the toxicity of “nonmetabolized” compounds prevails in preclinical models [21]. This review aims to show the state of the art about the consumption of NCS in children, as well as their benefits and consumption risks.

## 2. Methodology

Scientific searchers were used (PUBMED, Scopus, Scielo, and MEDLINE) to analyze articles that included keywords (noncaloric sweeteners/saccharin/cyclamate/acesulfame potassium/aspartame/sucralose/stevia/children) in English, Spanish, and Portuguese.

Evidence indicates that the consumption of NCS (diet products and snacks) between meals may offer an optimum benefit in this subgroup of the population and may reduce total caloric intake, while consumption with meals may result in compensation for the increase in food-related calorie intake [22]. The Academy of Nutrition and Dietetics states that NCS should not be used in children <2 years old and should be minimal or completely restricted during pregnancy and lactation, as the FDA has stated that use is unsafe [23]. Cyclamate and saccharin should be avoided, while another NCS can be used in moderate amounts or, even better, not used at all [24]. On the other hand, some studies [25–27] have associated the intake of sucralose and aspartame with migraine in adults. However, this type of studies is limiting to obtaining general conclusions, since only 3 clinical cases with positive association have been reported. Although it is important to analyze and report these investigations to generate possible safety signals, it would not be appropriate to issue a conclusive perspective based only on this data.

In a recent research which involved a sample of 100 children ages 3 to 15 years old, it was shown that only a small group (13 participants) had significant improvement in chronic head pain after exclusion of aspartame intake. The remaining participants associated headaches with other foods and/or additive [28]. Clear limitations of this research such as nonrandomized design and observational prospective cohort without control group prevent finding relevant information on this respect. However, this type of study opens the possibility of performing experimental designs with this hypothesis.

Children are especially important because they have a higher intake of food and drink per kilogram of their body weight [21]. A pediatric epidemiological study has found a positive correlation between the consumption of NCS contained in beverages and weight gain; however, conclusive data are still lacking [22]. In other context, recent studies with maximal electroshock seizure model (MES) demonstrated potential anticonvulsant effects of NCS such as acesulfame potassium, cyclamate, and saccharine; nevertheless this study was performed in animal, and it would be necessary to extrapolate it to humans [28].

## 3. Sweeteners and Dietary Intake

Drinks have been identified as an important source of NCS in the diet [29]; therefore NCS consumption estimates are usually based on artificially sweetened drinks or soft drinks. Consumption of artificially sweetened soda seems to be increasing in children, both with age and with time [30, 31]. Schoolchildren, especially the younger ones and of lesser weight, are a group that is usually studied because of the high probability of overcoming the daily intake (ADI) of each of the NCS. Evaluations carried out in the United Kingdom in the 1990s and 2003 indicated that ADI was exceeded by preschoolers aged between 1.5 and 4.5 years for two NCS (saccharin and cyclamate) [32].

However, it is currently unlikely that intake will exceed the ADI because of the large amount of NCS on the market, resulting in a reduction in the intake of certain NCS unless only one NCS is preferred. A study in Irish children determined the intake of 4 NCS. The results showed that the average daily intake for all four sweeteners (acesulfame K, aspartame, saccharin, and sucralose) was according to ADI (17–31%) [6].

A study in Chile [33] has shown that more than 90% of schoolchildren consume NCS but do not show that the intake exceeds the ADI; the high consumption of NCS is reflected mainly in the intake of products such as soft drinks, packaged juices, and powder juice; however, another study carried out in Argentine schoolchildren shows that 17% consume NCS and 1.5% of the population of children and adolescents surveyed (*n* = 190) exceed the ADI for cyclamate only through the consumption of soft drinks [15]. EFSA also concluded that ingestion of these NCS would not be considered a safety concern among this population group, which is consistent with results reported in other studies [34].

## 4. Sweeteners and Adipogenesis

Adipogenesis is the cellular process where precursor cells, mesenchymal stem cells, become an adipocyte cell. This process implicates that the precursors or preadipocyte undergo dramatic changes in phenotype and gene expression. Regulation of adipocyte differentiation relies largely on its surrounded environment with activation of specific genes like phosphorylated peroxisome proliferator-activated receptor *γ* (PPAR*γ*) and CCAAT/enhancer-binding protein *α* (C/EBP*α*), primary drivers of the adipogenic program that stimulate expression of terminal adipocyte genes such as fatty acid-binding protein 4 (FABP4) and GLUT4 [35].

Interestingly, many artificial sweeteners are pharmacologically active and can play a role in the regulation of the adipogenesis. Simon et al. [36] found that sweeteners such acesulfame K and saccharin stimulated the adipogenesis and inhibited the lipolysis in 3T3-L1 cells culture and these effects were independent of the classical sweet taste receptors, T1R2 and T1R3.

On the other hand, Pandurangan's group [37] showed that aspartame reduced 3T3-L1 adipocyte differentiation by downregulating p-PPAR*γ*, FABP4, and C/EBP*α*. In this study, 3T3-L1 cells were differentiated during 6 d in the absence and presence of aspartame and it was observed that aspartame could reduce significantly the lipid accumulation as evidenced by Oil Red O staining.

In other study, experiments with mice fed with D-xylose and with high fat diet resulted in the downregulation of adipogenesis-related genes in visceral adipose tissues. The authors concluded that D-xylose may play a role in the attenuation of the adipogenesis in vivo [38].

Regarding stevia, its effect in the adipogenesis process has not yet been studied; however, a study [35] reported that stevioside from* Stevia rebaudiana* has a significant action on 3T3-L1 insulin sensitivity, which meant an enhancement of glucose uptake, but at the same time an upregulation of genes related to cellular pathway elicited by insulin.

## 5. Sweeteners and Obesity

The consumption of sugary beverages shows a positive effect on adiposity [39]. However, associations are still being controversial [40]. Strong evidence supports the association of added sugars with the increased risk of cardiovascular disease in children through increased energy intake, increased adiposity, and dyslipidemia [41]. Substitution of caloric sweeteners (usually sugar) with low-calorie alternatives is a strategy that can help reduce energy consumption, facilitating weight loss and/or maintenance, and even prevention of weight gain [42–45].

In a study by de Ruyter et al. 641 schoolchildren with normal weight were randomly given 250 ml of sweetened drink or diet drink for a period of 18 months. The most important results were that the z-IMC increased by an average of 0.02 units and the body weight by 6.35 kg in the NCS group, while the sugar group increased z-IMC by 0.15 units and the body weight at 7.37 kg. The sum of the skinfolds, waist-height ratio, and fat mass also increased significantly in both groups but was lower in the NCS group [46]. On the other hand, in a randomized controlled trial, girls between 11 and 15 years old consumed a diet of 1500 kcal/day during 12 weeks. In one group, sugary drinks were allowed as a snack, while in the other group only diet soft drinks were allowed. There were no differences between groups for BMI change [47].

A recent meta-analysis conducted in randomized controlled trials and prospective cohort studies in both children and adolescents showed that NCS consumption exhibited a modest but significantly reduced body weight (−0.80 kg; IC of 95%  −1.17, 0.43), BMI (kg/m^2^): −0.24; IC of 95%  −0.41, −0.07), fat mass (−1.10 kg, IC of 95% CI −1.77, −0.44), and waist circumference (−0.83 cm, IC of 95%  −1, 29, −0.37). Among prospective cohort studies, NCS intake was not associated with body weight or fat mass but was significantly associated with a slightly higher BMI (0.03; IC of 95%: 0.01, 0.06) [48]. In a study by Katan et al. [49], it was found that the substitution of 250 ml per day of sugary drinks for diet drinks allowed a weight reduction per 1 kg in 18 months.

Various clinical studies have shown that sugar-free beverages reduce weight gain compared to sugar-sweetened beverages and besides NCS consumption allows better adherence to diet [39, 46–48], which could be an aid in the treatment for childhood and adolescent obesity [50–52]. A cross-sectional study that included 571 Chilean schoolchildren between 10 and 16 years old, of both sexes, found no association between NCS use and obesity risk [14].

On the other hand, three transversal studies, including 385 and 3311 children, showed positive association between the intakes of NCS and BMI [53]. Similar results were obtained with pregnant woman who ingested NCS, showing more probability of having babies with increased risk for later obesity or overweight. However, the limitation of the studies is that these were of observational type, and the findings do not necessarily imply a significant correlation between the intake of artificial sweeteners and weight gain [54, 55]. In a meta-analysis of intake of NCS that included 11.774 citations, 7 trials, 1003 participants, and 30 cohort studies (adults and adolescents) it was concluded that there is not enough evidence from randomized controlled trials to demonstrate the positive effect of NCS on controlling body weight. Findings of observational studies suggest that the continuous ingestion of NCS could be associated with BMI and cardiometabolic risk increase [56].

## 6. Other Possible Effects

A study that included 1988 girls showed that the consumption of soft drinks sweetened with NCS was positively associated with the risk of early menarche (RR of 1 serving/day: 1.43, 95% IC 1.08, 1, 88) and in particular aspartame (RR for the increase of 1-SD: 1.20, 95% IC: 1.10, 1.31) was positively associated with the risk of early menarche; the authors postulate that the mediator would be insulin [57]; however, caution should be exercised in interpreting the results for possible reverse causality. In contrast with these findings, a substantial body of scientific evidence supports that NCS do not have any effect on menarche or delayed puberty in girls [58, 59].

Until now, it is not totally clear if there are correlational associations between NCS intake and raised risk illness in children; however different studies demonstrate that NSC could be a better option than sugar-sweetened beverage; it does not necessarily imply that NCS is totally harmless to human health. In this context, it is believed that it is possible that the chronic intake of NCS in children could produce a negative effect on health; for example, the exposure to artificial sweeteners during infancy can affect the adult eating habits and could cause an increase in the consumption of sugars throughout adulthood [60]. In addition to this premise, the underlying psychological factors are relevant in the food industry, as they influence the selection of food by the child population. Minimum standards on nutritional content of these products are required to ensure that they comply with the nutritional recommendations of this age group [61].

Other aspect of NCS is about the rewarding postintake feedback, because NCS produce sweet taste without giving calories. Studies of Frank et al., [39] and Smeets et al., [62] demonstrated that sucrose, but not NCS, could elicit the reward pathway. Moreover, NCS is not able to produce postprandial release of insulin, GLP-1, or GIP [63], which can finally produce a subsequent feeling of dissatisfaction with additional food intake compensation. Interestingly, Wang et al., studying sucralose demonstrated that chronic consumption of a sweet/energy imbalanced diet promoted an increase in food intake in mammals through a NPY-dependent mechanism, triggering a conserved neuronal response in fasting and increasing motivation to eat [64]. Likewise, certain studies have related a negative impact of NCS on gut microbiota [65, 66] which, in turn, could be a factor towards the development of obesity and insulin resistance. Particularly, the artificial sweetener saccharin could alter gut microbiota and induce glucose intolerance, raising questions about the contribution of artificial sweeteners to the global epidemic of obesity and diabetes. In this regard, a study found that consumption of a nonnutritive sweetener containing 1% sucralose impaired the growth of gut bacteria in rats [67]. In addition, a recent study found that noncaloric artificial sweeteners, such as saccharin, impaired glucose tolerance by modulating the composition of gut bacteria [68]. A recent research found that Ace-K consumption perturbed gut microbiome of CD-1 mice after a 4-week treatment [69]. The observed body weight gain shifts in the gut bacterial community composition, the enrichment of functional bacterial genes related to energy metabolism, and the fecal metabolomics changes were highly gender-specific with differential effects observed for males and females. However, the number of specific studies on the microbiome is very limited, and currently research in this area with specific effects in childhood has not yet been developed.

In another context, one of the possible benefits of sweetener consumption in children is its anticariogenic effect. The sugars in sticky foods consumed between meals were associated with high cariogenic activity. These findings stimulated the investigation of nonacidogenic sugar substitutes (sweeteners) that do not cause pH drops in dental plaque [70]. Fermentable carbohydrates of the diet give place to acidic material that destroys the hard dental tissue, causing cavities. In the infant population, in addition to fluoride deficiency, cavities are still frequent because after the intake of sweet foods there is usually no adequate oral hygiene [71]. On the other hand, in infants, the use of juices or other sugary beverages rich in fructose or sucrose, for long time, increases the risk of dental damage. Among the sweeteners that have been declared with healthy properties to avoid this pathology, there are the polyalcohols of sugar or the sucralose among others [72]. The risks and benefits of NCS are shown in Table 1.

Finally, it is important to note that NCS are used clinically in products for pediatric use, for example, in protein substitution formulas in which sucralose is added for patients with phenylketonuria and other inborn errors of metabolism [8]. Similarly, this NCS has been used to sweeten specific enteral formulas for patients with diabetes, with favorable glycemic and insulin responses in healthy and diabetic adults. However, it is necessary to conduct scientific research in this specific field to evaluate the metabolic effect of NCS on health, especially in children and adolescents with diabetes [73–75].

## 7. Conclusions

It is imperative that health professionals judiciously and individually evaluate the overall benefits and risks of NCS use in consumers before recommending their use. Different subgroups of the population incorporate products containing NCS in their diet with different objectives; however, these types of additives should be used in children belonging to earlier age groups, only as a dietary alternative when other forms of prevention in obesity are not sufficient. Since the use of artificial sweeteners has increased in the last decades, it is clear that it is crucial to perform more studies in order to determine more accurately the effect of NCS on health, in order to elaborate recommendations with scientific support for the prevention and treatment of children obesity.

## Figures and Tables

**Table 1:** Number of Publications that show risks versus benefits of the use of artificial sweeteners and number of studies in humans and animal models.

Year of publication	Number of study benefits	Number of study risks	Total	Number of animals models	Number of human studies	Total
	16	21		11	26	
2000–2005	2	4	6	0	6	6
2006–2012	6	2	8	5	3	8
2013–2017	8	15	23	6	17	23

Total	**16**	**21**	**37**	**11**	**26**	**37**
